# Preliminary Evidences of Biofortification with Iodine of “Carota di Polignano”, An Italian Carrot Landrace

**DOI:** 10.3389/fpls.2018.00170

**Published:** 2018-02-12

**Authors:** Angelo Signore, Massimiliano Renna, Massimiliano D'Imperio, Francesco Serio, Pietro Santamaria

**Affiliations:** ^1^Department of Agricultural and Environmental Science, University of Bari Aldo Moro, Bari, Italy; ^2^Institute of Sciences of Food Production, National Research Council, Bari, Italy

**Keywords:** *Daucus carota* L., Polignano carrot, multi-colored roots, iodine RDA, soil-less

## Abstract

The “*Carota di Polignano*” (Polignano Carrot – PC, *Daucus carota* L.) is a multi-colored landrace, cultivated in the Southern Italy, whose colors range from yellow to purple. Iodine is an essential micronutrient for humans, since it is a key component of thyroid hormones, which regulate the growth and development of the human body. The main source for iodine assumption is represented by diet, but its concentration in the vegetables is usually limited with respect to human needs. To this purpose, two experimental trials (in open field and in greenhouse with a soil-less system) were carried out to enrich PC with iodine. Three levels of iodine (control treatment, C – 0 mg·L^−1^; low, L – 50 mg·L^−1^; and high, H – 500 mg·L^−1^), distributed with foliar spray fertilizations (in both open field and greenhouse) or with nutrient solution (in greenhouse, at the level of 50 mg·L^−1^) in the form of KIO_3_ were compared. In open field, the H treatment showed a biofortification that was double and triple respect to L and C treatments, respectively, without influencing color and biometric parameters, such as the fresh and dry weight of roots and DM percentage. In greenhouse, the biofortification done with foliar spray fertilization followed the same trend of open field, while the biofortification by means of nutrient solution was more effective but reached very high levels that had toxic effects on the plants and could be too high for human nutrition. However, the concentrations of iodine into biofortified carrots in open field can allow to satisfy the recommended daily allowance (RDA) by consuming 100 and 200 g of fresh product for the treatment H and L, respectively. Regarding the greenhouse biofortification, the RDA would be satisfied by consuming 200 g of fresh carrots (with the high level of foliar fertilization).

## Introduction

Italy is one of the most important vegetable-producing country in Europe, and the Puglia Region (Southern Italy), that forms about 22% of the Italian total vegetable-growing area, is the most important region in Italy for open field crops, with more than 100,000 hectares (Istituto Nazionale di Statistica, 2017^1^). Because of its particular conformation and position, the Puglia region holds a great heritage of agro-biodiversity, with particular reference to vegetables. Unfortunately, such agro-biodiversity has been partially lost, due to several factors (Elia and Santamaria, 2013; Signore, 2016). To counteract such loss, in a context of a project about agro-biodiversity, the Puglia Region Administration undertook several initiatives, with the aim to identify, protect and recover several landraces of vegetables at risk of genetic erosion (Renna et al., 2014). Among such landraces, there is a carrot called, in Italian, *Carota giallo-viola di Polignano* (yellow-purple carrot of Polignano—the municipality where it is located), a multicolored (ranging from yellow to purple tone) landrace of *Daucus carota* (L.), from here onward named PC, that has been previously studied and characterized for some parameters, such as the compositional and antioxidant profiles (Cefola et al., 2012). However, one of the main purposes of the Puglia Region Administration plan is to valorize such landrace(s), in order to push more and more farmers to cultivate them, and realize a full recovery of the agro-biodiversity. However, such valorization passes not only from the farmers, but also from consumers. Since the PC has higher prices on the market, up to 2–3-fold the “commercial” carrot, to stimulate its consumption is crucial to find an added value that could push the consumers to prefer the PC to the “commercial” one. An added value for a product may be represented by its nutritional aspects, either already present in its composition, or subsequently added.

A way to realize such added valued may be represented by the biofortification, a technique that consists in adding some (micro)nutrient(s), beneficial for human health, to food. From this point of view, iodine it is a perfect example, because is responsible, together with other minerals such as vitamin A and iron, of the “hidden hunger,” defined by the WHO as “a lack of vitamins and minerals” (World Health Organization, 2004^2^). Iodine is an essential trace element for human, since it is a fundamental component of thyroid hormones that regulate the growth and development of the body, and its deficiency may strongly affect the functionality of thyroid, by the means of two iodine containing-hormones: triiodothyronine (T3) and thyroxine (T4). From this point of view, the iodine is a rate-limiting element for the synthesis of such hormones.

According with White and Broadley (2009), 30–38% of the world's population has insufficient iodine intake and live with risk for iodine deficiency, and associated iodine deficiency disorders (IDD). According to Global Iodine Network (2017)^3^, such deficiency is present even in developed countries: Italy for example has an insufficient iodine intake. The iodine deficiency has been associated with several diseases, such as mental impairment and goiter in older children and adults (de Benoist et al., 2008) and complications with pregnancy, during which inadequate iodine intake may lead to irreversible brain damage to the fetus (World Health Organization, 2007).

For iodine, the Recommended Daily Allowance (RDA) value changes according to the Country, the Organization that suggests the RDA, the age and other factors such as, for example, being a pregnant or breastfeeding woman, with the values that normally range from 90 to 290 μg (Zimmermann, 2017). The problem of IDD is even more serious considering that the consumption of one of the most important sources for iodine, milk, has decreased since the 1950s, even if it has remained relatively steady in recent years, and milk alternatives have negligible iodine content, therefore are not appropriate substitutes in terms of iodine provision (Bath et al., 2017). To counteract the IDD, one of the most effective way is the iodization of kitchen salt but, even so, a third of the global population is still unprotected from iodine deficiency (World Health Organization, 2005; Gunnarsdottir and Dahl, 2012; European Food Safety Authority, 2014). Moreover, the iodine has low stability in salt and losses occur in the several steps of production, packaging, transportation and processing, hence the total amount of iodine lost from salt may reach 90%, with the cooking process that may contribute on average to 20% of such losses (Winger et al., 2008). Such losses can occur even in the food: for example the biofortified carrots may lose around 55% of their iodine content during the boiling process (Comandini et al., 2013). Besides, the excessive consumption of salt has some drawbacks, because it may cause problem of hypertension. According to Zimmermann and Andersson (2012), the current salt intake in children is unnecessarily high and is very likely to predispose children to develop hypertension later.

Thus, to overcome the salt-related problems, the biofortification should be done with food, specifically vegetables, that do not have the salt side effects, so they may be used as a vector for this mineral in the diet, since many of such products are consumed raw (Haldimann et al., 2005) and the iodine losses are usually negligible. Several vegetable crops can store iodine such as lettuce (Cerretani et al., 2014), spinach (Dai et al., 2006), tomato (Landini et al., 2011; Kiferle et al., 2013; Smolen et al., 2015) and carrot (Dai et al., 2004; Hong et al., 2008; Comandini et al., 2013; Smolen et al., 2016). Starting from the above premises, the scopes of our research were to: (i) enrich a local landrace of carrot with iodine, both in a traditional cultivation system (open field) and with an advanced system (soil-less system) and (ii) evaluate the effect of iodine concentration on the other quality parameters of the carrot root.

## Materials and methods

### Open field—crop system and treatments

The trial was carried out between October 2013 and April 2014, in a field located in Polignano a Mare, Southern Italy (41.011111, 17.189694).

The soil of the experimental field, which distance from the sea was about 400 m, was basically sandy and had a concentration of total nitrogen of 1.3‰, while the concentration of organic matter was 2.33%.

The temperature ranged from 3.5° to 25°C and the percentage of relative humidity from 25 to 99% during the crop cycle (minimum/maximum respectively—data not shown).

The experimental treatments were arranged in a completely randomized design with three replications. Every plot was a square with a side of 3 m width having a border zone of 1 m. The sowing was done on October 15, by putting the seeds in continuous manner on the row (30–40 seeds·m^−1^). The distance between rows was 0.35 m, resulting in a final density of 70–100 plants·m^−2^, according with the common practice (for the details regarding sowing and other crop techniques, see the video in the “Supplementary Material” section).

The fertilization of the field was not necessary, since local farmers apply the agronomic principle of crop rotation, thus soil fertility remaining from the previous crop is sufficient to satisfy the needs of the PC (Renna et al., 2014).

The iodine biofortification was realized by spraying iodine on the leaves in form of potassium iodate (KIO_3_, Sigma-Aldrich ACS reagent, purity 99.5%). Three different levels of iodine were compared, namely:

- 0—control: (no foliar biofortification—no iodine was added);
- FB-L (foliar biofortification—low level): the iodine concentration was 50 mg·L^−1^ (0.394 mM) for every application;
- FB-H (foliar biofortification—high level): the iodine concentration was 500 mg·L^−1^ (3.94 mM) for every application.

In total, four foliar applications were realized: the first one was realized on January 30, at the plant stage of full vegetative growth (30 cm height), while the other applications were distributed fortnightly, the latest one with the roots fully developed. For every treatment, the volume used was 1 L·m^−2^. The irrigation was done by sprinkling.

The Harvest Index (HI) was calculated with the following formula:

$$\begin{array}{l} FW\left(roots\right)/FW\left(roots\right)+FW\left(aerial\;biomass\right) \end{array}$$

where FW = fresh weight.

### Greenhouse—crop system and treatments

The trial was carried out at the “La Noria” experimental farm (Institute of Sciences of Food Production of the National Research Council) located in Mola di Bari (41.06214, 17.06685—Southern Italy), in a polymetacrylate non heated greenhouse with a maximum height of 4.5 m. The temperature ranged from 4° to 34°C and the percentage of relative humidity from 25 to 99% during the crop cycle (minimum/maximum respectively—data not shown).

Plants were arranged on 12 aluminum benches (length 6 m, width 0.26 m, 1% sloped) on which were positioned 20 pots for bench, each of which had a volume of 8.5 L. The distances of the pots on the rows and the distance between the rows were 0.2 m and 0.33 m, respectively. The treatments were arranged in a randomized block design, with two replications and four benches that served as guard rows, two external and two between the blocks. Every pot contained perlite as substrate (AGRILIT 3, Perlite Italiana) and, in the upper part, a peat layer of 0.5 cm that was positioned in order to ensure a uniform distribution of the watering in the first phases of seeds germination. The sowing was realized on November 18, by putting 4 to 6 seeds per hole, in four holes arranged according to the vertexes of a square. After the complete seedling emergence, at the stage of 1st–2nd true leaf, a thinning was done to have four plants per hole.

On December 27, the fertigation with nutrient solution (NS) was started. The NS, which pH and electric conductivity (EC) values were 5.7 and 3.4 dS·m^−1^, respectively, had the following composition: N (14 mM), P (1 mM), K (6 mM), Mg (2 mM), Ca (4 mM), S (2 mM), Fe (20 μM), Mn (5 μM), Zn (2 μM), B (25 μM), Cu (0.5 μM), and Mo (0.1 μM). Fertigation was realized by using a pressure compensating emitter per pot, with a flow of 8 L·h^−1^. The NS was managed in open cycle, and the frequency of irrigations was adjusted to maintain the drainage percentage between 30 and 50%. The experimental treatments were differentiated on March 7, by means of potassium iodate (KIO_3_, Sigma-Aldrich ACS reagent, purity 99.5%). The treatments were the following:

- 0—control: (no foliar biofortification—no iodine was added);
- FB-L (foliar biofortification—low level): the iodine concentration was 50 mg·L^−1^ (0.394 mM);
- FB-H (foliar biofortification—high level): the iodine concentration was 500 mg·L^−1^ (3.94 mM);
- NS-L (NS biofortification—low level): the iodine concentration was 50 mg·L^−1^ (0.394 mM);

The foliar applications were repeated fortnightly (three applications in total—at the same plant stage of open field experiment), while the iodine into the NS was provided continuously until the harvest.

### Measurements and analysis

In the second experiment, we have analyzed the three colors of the carrots, namely, yellow, orange and purple.

The dry matter (DM) was determined after drying until constant weight in a forced-draft oven at 65°C, for at least 72 h.

The concentration of the nitrates was determined by ion chromatography (Dionex model DX120; Dionex Corporation, Sunnyvale, CA, USA) with a conductivity detector, using the pre-column IonPack AG14 and the column of separation IonPack AS14 (Signore et al., 2016).

#### Extraction of inorganic iodine

For the extraction and quantification of inorganic iodine, the roots were separated into the different colors (yellow, orange, and purple).

The inorganic iodine determination was determined using the protocol by Perring et al. (2001). Briefly, for analysis of Iodine content, 2–3 g of lyophilized carrots samples were treated with 50 mL of hot water (60°C) and stirred for 30 min at room temperature. After extraction, the samples were diluted and filtered by using Whatman filter paper followed by 0.2 μm membrane filter. The resulting solutions were used for quantification of inorganic iodine content.

#### Quantification of inorganic iodine content

The analysis of inorganic iodine contents was determined using the spectrophotometric methods described by Perring et al. (2001). Briefly, iodate standard solution and the extracts samples (100 μg·L^−1^) were treated with 1 mL of KSCN (0.023% m/v), 2 mL of NH_4_Fe(SO_4_)_2_ (7.7% m/v) in 2.4 M HNO_3_ and 2 mL of NaNO_2_ (0.02% m/v). The solutions were mixed and incubated in water bath at 60 ± 2°C for 1 h and subsequently incubated for 10 min in a water–ice mixture in order to stop the colorimetric reaction. Each solution was read at 454 nm. The quantification of inorganic iodine in carrots was determined by interpolation with a calibration curve, previously made (0–12 μg/L; *R*^2^ = 0.9895).

### Statistical analysis

The statistical analysis was performed with the Statistical Analysis System software SAS (Cary, NC, USA) using the GLM (General Linear Model) procedure for the analysis of variance.

For all the parameters, the comparison between the means point was performed by calculating the least significant difference (LSD, *P* = 0.05).

## Results

The biofortification did not affect any of the biometric parameters in the open field experiment (Table 1) as well as in greenhouse (Table 2). The FW of leaves and roots (Table 2) and the harvest index (data not shown) were influenced neither by the type of biofortification nor by the level of iodine used.

**Table 1:** Iodine concentration in the roots, fresh and dry matter weight in leaves and roots—open field.

**Biofortification**	**Iodine (μg·100 g FW^−1^)**	**Leaves**		**Roots**	
		**Fresh weight (g)**	**Dry weight (g)**	**Fresh weight (g)**	**Dry weight (g)**
0	59 c (±5.98)	219 (±10.20)	25.2 (±0.82)	115 (±11.71)	9.86 (±0.88)
FB-L	89 b (±10.63)	209 (±11.61)	27.6 (±2.18)	111 (±5.24)	10.13 (±0.33)
FB-H	174 a (±10.68)	217 (±1.10)	27.1 (±2.23)	109 (±6.73)	9.43 (±1.17)
Significance^a^	^***^	ns	ns	ns	ns

a Significance of F: ns, not significant for P ≤ 0.05;

*** *, significant for P ≤ 0.001. Different letters indicate statistically significant differences at P = 0.05. Number of observations (replications) = 3*.

**Table 2:** Fresh weight and dry matter percentage in leaves and roots and iodine concentration in roots—greenhouse.

**Biofortification**	**Leaves**		**Roots**		**Iodine (μg·100 g FW^−1^)**
	**Fresh weight (g)**	**Dry matter (%)**	**Fresh weight (g)**	**Dry matter (%)**	
0	258 (±50.1)	7.95 (± 7.9)	66.9 (± 21.4)	7.16 (± 0.8)	1.2 c (±0.04)
FB-L	221 (±50.7)	8.81 (± 8.8)	53.6 (± 15.7)	7.35 (± 1.2)	35.4 bc (±2.53)
FB-H	283 (±146.5)	7.94 (± 7.9)	76.2 (± 28.7)	7.04 (± 0.5)	75.1 b (±1.11)
NS-L	280 (±60.7)	9.08 (± 9.1)	88.7 (± 28.8)	8.10 (± 0.8)	896.0 a (±43.5)
Significance^a^	ns	ns	ns	ns	^***^

a Significance of F: ns, not significant for P ≤ 0.05;

*** *, significant for P ≤ 0.001. Different letters indicate statistically significant differences at P = 0.05. Number of observations = 6 (2 replications and three colors)*.

**Figure 1:**
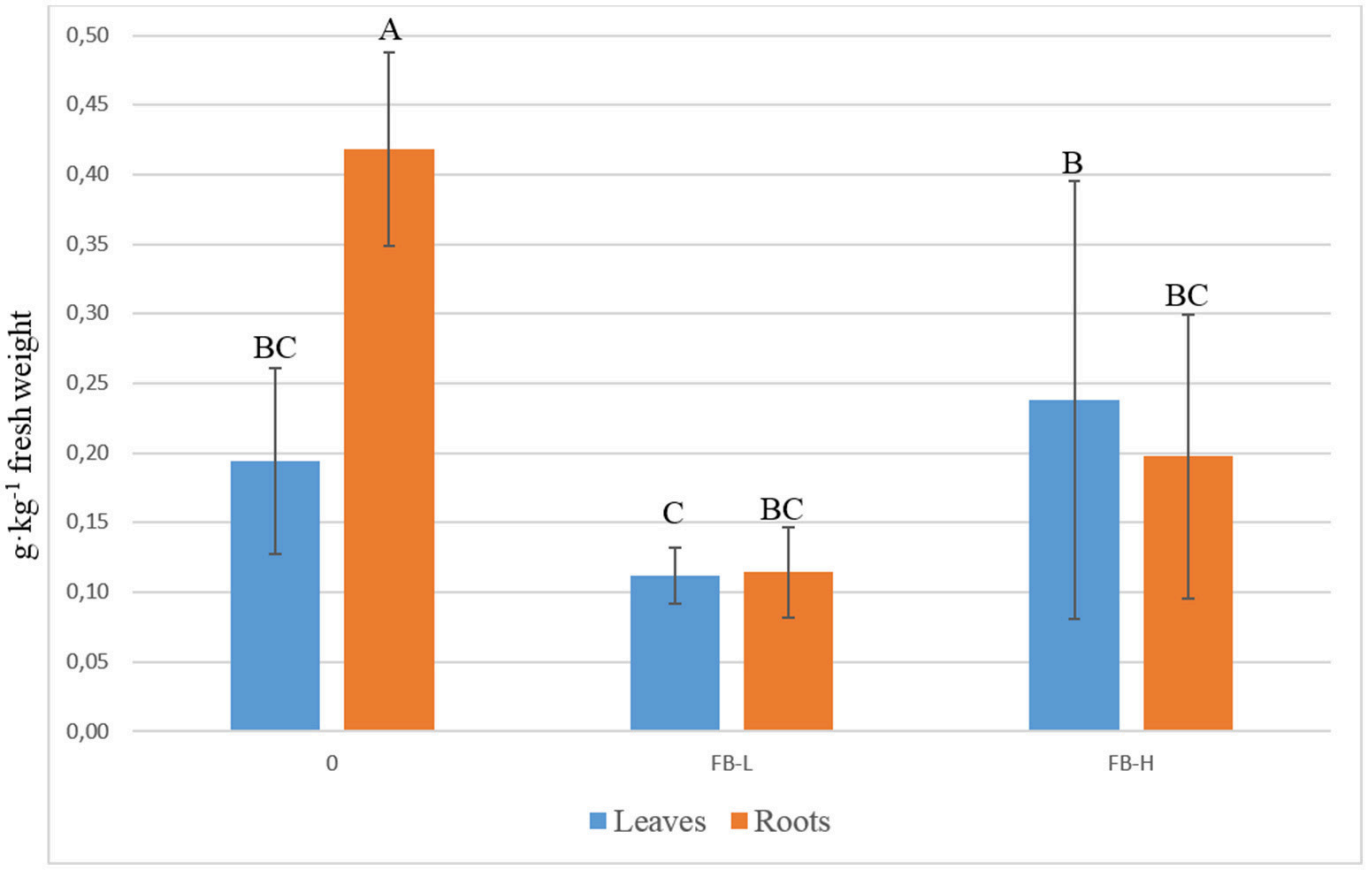
Concentration of the nitrates according with the part of considered plant and as a function of the experimental treatment—open field. Vertical bars represent the standard deviation. Different letters indicate that mean values are significantly different, according to the LSD method *P* = 0.05.

The nitrate (${\text{NO}}_{3}^{-}$) concentration on DM basis was not influenced by the biofortification, but the treatments acted jointly with part of the plant in determine some significant differences (Figure 1), even if the ${\text{NO}}_{3}^{-}$ concentration measured on the fresh basis was not different (data not shown). Into the roots, the ${\text{NO}}_{3}^{-}$ concentration was higher in the control treatment with respect to the biofortified ones (Figure 1), while in the leaves the only significant difference was between FB-L and FB-H, with the latter that produced a ${\text{NO}}_{3}^{-}$ concentration 112% higher with respect to FB-L (Figure 1). The difference in ${\text{NO}}_{3}^{-}$ concentration between leaves and roots is clearly visible in the control treatment, but the biofortification treatment has flattened such difference (Figure 1).

The biofortification has increased the iodine concentration of the roots in both open field (51% in FB-L treatment and 194% in FB-H treatment with respect to the non-biofortified carrots—Table 1) and greenhouse experiment (but only when the iodine was applied by the means of the NS—Table 2), even if the different colors of the carrot did not produce any significant difference (data not shown).

## Discussion

**Figure 2:**
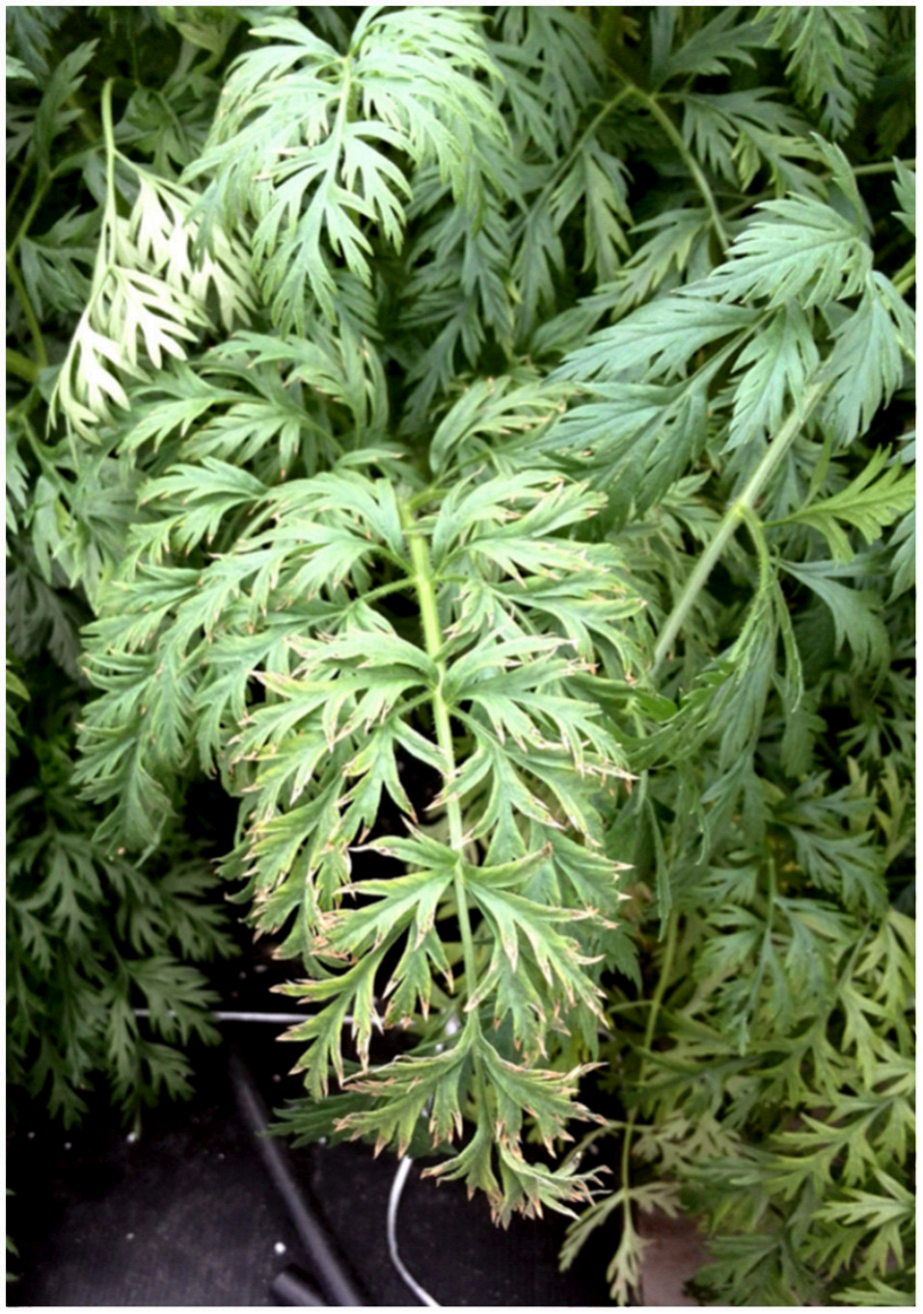
Iodine injuries on the outer margin of the leaves.

In our experiments, the biometric parameters were not influenced by the biofortification treatments, not in open field nor in greenhouse (Tables 1, 2) in agreement with the results reported by Smolen et al. (2014). Generally, high concentration of iodine may lead to a detriment of biomass even at low rates, as reported by Caffagni et al. (2011), whose iodine levels ranged from 0 to 23 mM, but this was not our case. Such result is not surprising, since carrot is reported to have a good tolerance to high levels of iodine (Hong et al., 2008). However, when the iodine was distributed via the NS, we observed a negative effect on the leaves (Figure 2) i.e., necrosis on the leaves, in particular in the outer margins of the older ones, without any effect on the roots. As reported by other Authors, such negative aspect is usually more pronounced into the shoot than in the “below ground” organs (Hong et al., 2008; Caffagni et al., 2011), probably because iodine is transported in the xylem rather than in the phloem (Herrett et al., 1962; Mackowiak and Grossl, 1999; Mackowiak et al., 2005). The magnitude of the injuries on the leaves depends on several factors such as the species, the chemical form of iodine used, the method of iodine application and the crop environment (open field or greenhouse) (Mackowiak et al., 2005; Weng et al., 2008a,b; Caffagni et al., 2011). In our case, this negative effect would indicate that, in a soil-less system, the iodine distributed via the NS accumulates continuously and cumulatively into the leaves tissues; therefore, the concentration of iodine that should be used within the NS has to be previously considered, by both decreasing its concentration into the NS and avoiding its application during every single fertigation.

The nitrates concentration on DM basis was not influenced by the biofortification, but the iodine concentration and the organ considered (leaved and roots) modulated the final concentration, as reported in Figure 1, and is clearly visible that the difference in nitrate concentration between leaves and roots has been flattened by such interaction. Such result may be explained with the existing linkage between the metabolism of ${\text{IO}}_{3}^{-}$ and the reduction of ${\text{NO}}_{3}^{-}$ into the plant tissue (Wong and Hung, 2001; Hung et al., 2005; Smolen et al., 2014), even if the data regarding such interaction are sometimes ambiguous (Gonda et al., 2007). Blasco et al. (2010) in and experiment with lettuce, compared three level (20, 40, and 80 μmol·L^−1^) and two forms of iodine (I^−^ and ${\text{IO}}_{3}^{-}$), and found that the application of I^−^ (as KI), at levels of at least 20 μM, reduced both ${\text{NO}}_{3}^{-}$ accumulation and NR activity (NR_act_) in leaves of lettuce. Conversely, the same levels of iodine, but in the forms of ${\text{IO}}_{3}^{-}$ (as KIO_3_), increased NR_act_, but did not produce any influence on ${\text{NO}}_{3}^{-}$ concentration in lettuce leaves. In our case, the concentration of ${\text{NO}}_{3}^{-}$ into the roots of the biofortified treatments (Figure 1) was not different from that into the leaves. In the control treatment, the NR_act_ into the roots was probably involved mainly in the reduction of ${\text{IO}}_{3}^{-}$ to I^−^ at the expense of the ${\text{NO}}_{3}^{-}$ reduction process, while in the biofortified treatments the NR_act_, which value was probably higher with respect to the control treatment, consistently with the results of Blasco et al. (2010), could cope with the reduction of both ${\text{IO}}_{3}^{-}$ to I^−^ and ${\text{NO}}_{3}^{-}$ to ${\text{NO}}_{2}^{-}$. The higher ${\text{NO}}_{3}^{-}$ concentration in the leaves of the FB-H with respect to the FB-L treatment, could be explained by the level of iodine used: in fact, as reported by Blasco et al. (2010), the NR_act_ rose up to a certain concentration of iodine, then was reduced, hence inducing an higher concentration of nitrates. Moreover, the carrot accumulates more iodine into the shoot than the root (Hong et al., 2008), so this could have led to a greater suppression of NR_act_ in the leaves.

The iodine concentration of carrot roots was increased by the biofortification treatments both in open field (174, 89, and 59 μg·100 g of FW^−1^ for FB-H, FB-L and the control, respectively) and in greenhouse, but in this case only with the iodine applied by the means of the NS (Table 2), consistently with what has been reported by other authors (Dai et al., 2004; Hong et al., 2008; Smolen et al., 2014, 2016), even if the different colors of the carrot did not produce any significant difference (data not shown). Comparing the same levels of iodine in the FB treatments, the biofortification in the greenhouse seems to be less efficient than in open field (Tables 1, 2, respectively): however, such differences were almost surely due to the number of foliar biofortification applications. Indeed, we did four iodine application in open field, and three in the greenhouse, because in the latter there was a sudden flowering of the plants due the abnormal high temperatures reached (with maximum values higher than 30°C starting from February—data not shown). Interestingly, the iodine concentration of the carrots from the control treatment (without biofortification) in open field, showed an iodine concentration of 59 μg·100 g FW^−1^ (Table 1): such concentration is surely due to the small iodine concentration contained in both the irrigation water and in the soil (15 μg·L^−1^ and 2.5 mg·kg^−1^, respectively—data not shown). In open field, the iodine values in the carrots would allow to satisfy, or slightly exceed, the RDA of an adult (150 μg·day^−1^—European Food Safety Authority, 2014) by consuming 100 and 200 g of fresh product for the FB-H and FB-L treatment, respectively, in agreement with the values reported by Smolen et al. (2016). This is an important point, since the carrots may be consumed either raw (they keep almost all the iodine) or cooked. The cooking process may diminish the iodine availability in biofortified foods depending on the cooking method (Winger et al., 2008; Cerretani et al., 2014). On the other side, the level of iodine reached in the NS treatment (Table 2) was too high for both the plants and for human nutrition purposes. That means that, in a soil-less system, the dosage of the iodized fertilizer should be strictly optimized: indeed, a high dosage may disrupt the normal plant growth and reduce the efficiency of iodized fertilizer usage (Piatkowska et al., 2016). Such aspect was highlighted by Blasco et al. (2010), who reported that a bottleneck in the biofortification process is the need to increase the iodine concentration without any adverse effect on the plant growth. From the human health point of view, the iodine level in the NS treatment could be dangerous for human health, because the iodine toxicity may cause a wide spectrum of thyroid disorders, that may range from hyperthyroidism to hypothyroidism (Comandini et al., 2013; Cerretani et al., 2014), so further research are needed to tailor the amount of iodine in the NS for carrot biofortification.

Eventually, we choose the carrot as target for iodine biofortification because increasing the content of iodine in carrot instead of other vegetables has another positive aspect: it contains high level of β-carotene, a precursor of vitamin A, that has been found to have a positive effect on thyroid function (Zimmermann, 2007). Moreover, we did consider the biofortification of local landraces of carrot because, according to Cefola et al. (2012), they have a higher nutritional value with respect to commercial ones, and the need to increase the food production at worldwide level cannot be separated from the protection of biodiversity and traditional food and practices (Caffagni et al., 2011) since, as stated by Johns and Eyzaguirre (2007), “food biofortification, in order to have a positive impact, must be complemented both with conservation and greater use of biodiversity.”

## Conclusions

The IDD is a major problem even in the developed countries, and biofortification with iodine is a good alternative to iodized salt to cope with such problem. Our results showed that the iodine biofortification in the carrot root can be done via foliar treatments or fertigation but, in the latter case, further studies are needed to tailor the concentration of iodine and avoiding levels that may be harmful for the plants and human health. However, the iodine concentration reached with the foliar treatments would ensure an adequate RDA by simply consuming 150 g of product fresh weight. Since we found, from our previous study, that the qualitative profile of the “Polignano carrot” is really interesting, further studies are needed to clarify the effect of iodine biofortification on β-carotene contents, antioxidant activity, total carotenoids and total phenols.

## Author contributions

AS: Substantial contributions to the conception or design of the work; drafting the work; final approval of the version to be published; agreement to be accountable for all aspects of the work in ensuring that questions related to the accuracy or integrity of any part of the work are appropriately investigated and resolved. MR: revised the article critically; final approval of the version to be published; agreement to be accountable for all aspects of the work in ensuring that questions related to the accuracy or integrity of any part of the work are appropriately investigated and resolved. MD: Performed the analysis of iodine; revised the article critically; final approval of the version to be published. FS: Interpretation of data; revised the article critically; final approval of the version to be published; agreement to be accountable for all aspects of the work in ensuring that questions related to the accuracy or integrity of any part of the work are appropriately investigated and resolved. PS: Substantial contributions to the conception or design of the work; analysis and interpretation of data; final approval of the version to be published; agreement to be accountable for all aspects of the work in ensuring that questions related to the accuracy or integrity of any part of the work are appropriately investigated and resolved.

### Conflict of interest statement

The authors declare that the research was conducted in the absence of any commercial or financial relationships that could be construed as a potential conflict of interest.

## Footnotes

^1^Superfici Agricole Regione Puglia. http://agri.istat.it/—last access 19/12/2017.

^2^http://whqlibdoc.who.int/publications/2004/9241546123.pdf—last access 19/12/2017

^3^http://www.ign.org/cm_data/IGN_Global_Map_AllPop_30May2017.pdf—last access 19/12/2017

## References

[B1] Bath S. C. Hill S. Goenaga Infante H. Elghul S. Nezianya C. J. Rayman M. P. (2017). Iodine concentration of milk-alternative drinks available in the UK in comparison with cows' milk. Bri. J. Nutr. 20, 1–8. 10.1017/S0007114517002136 PMC5650045 28946925

[B2] Blasco B. Rios J. J. Cervilla L. M. Sánchez-Rodríguez E. Rubio-Wilhelmi M. M. Rosales M. A. (2010). Photorespiration process and nitrogen metabolism in lettuce plants (*Lactuca Sativa* L.): induced changes in response to iodine biofortification. J. Plant Growth Reg. 29, 477–486. 10.1007/s00344-010-9159-7

[B3] Caffagni A. Arru L. Meriggi P. Milc J. Perata P. Pecchioni N. (2011). Iodine fortification plant screening process and accumulation in tomato fruits and potato tubers. Comm. Soil Sci. Plant Anal. 42, 706–718. 10.1080/00103624.2011.550372

[B4] Cefola M. Pace B. Renna M. Santamaria P. Signore A. Serio F. (2012). Compositional analysis and antioxidant profile of yellow, orange and purple *Polignano carrots*. Italian J. Food Sci. 24, 284–291.

[B5] Cerretani L. Comandini P. Fumanelli D. Scazzina F. Chiavaro E. (2014). Evaluation of iodine content and stability in recipes prepared with biofortified potatoes. Int. J. Food Sci. Nutr. 7486, 1–6. 10.3109/09637486.2014.917155 24828007

[B6] Comandini P. Cerretani L. Rinaldi M. Cichelli A. Chiavaro E. (2013). Stability of iodine during cooking: investigation on biofortified and not fortified vegetables. Intl. J. Food Sci. Nutr. 64, 857–861. 10.3109/09637486.2013.798270 23701028

[B7] Dai J. L. Zhu Y. G. Huang Y. Z. Zhang M. Song J. L. (2006). Availability of iodide and iodate to spinach (*Spinacia Oleracea* L.) in relation to total iodine in soil solution. Plant Soil 289, 301–308. 10.1007/s11104-006-9139-7

[B8] Dai J. L. Zhu Y. G. Zhang M. Huang Y. Z. (2004). selecting iodine-enriched vegetables and the residual effect of iodate application to soil. Biol. Trace Elem. Res. 101, 265–276. 10.1385/BTER:101:3:265 15564656

[B9] de Benoist B. McLean E. Andersson M. Rogers L. (2008). Iodine deficiency in 2007: global progress since 2003. Food Nutr. Bull. 29, 195–202. 10.1177/156482650802900305 18947032

[B10] Elia A. Santamaria P. (2013). Biodiversity in vegetable crops: a heritage to save. The case of the puglia region. Italian J. Agron. 8, 21–34. 10.4081/ija.2013.e4

[B11] European Food Safety Authority (2014). Scientific opinion on dietary reference values for iodine. EFSA J. 12, 1–57. 10.2903/j.efsa.2014.3660

[B12] Gonda K. Yamaguchi H. Maruo T. Shinohara Y. (2007). Effects of iodine on growth and iodine absorption of hydroponically grown tomato and spinach. Hort. Res. Japan 6, 223–227. 10.2503/hrj.6.223

[B13] Gunnarsdottir I. Dahl L. (2012). Iodine intake in human nutrition: a systematic literature review. Food Nutr. Res. 56, 19731. 10.3402/fnr.v56i0.19731 23060737 PMC3468836

[B14] Haldimann M. Alt A. Blanc A. Blondeau K. (2005). Iodine content of food groups. J. Food Comp. Anal. 18, 461–471. 10.1016/j.jfca.2004.06.003

[B15] Herrett R. A. Hatfield H. H. Crosby D. G. Vlitos A. J. (1962). Leaf abscission induced by the iodide ion. Plant Physiol. 37, 358–363. 10.1104/pp.37.3.358 16655658 PMC549793

[B16] Hong C. L. Weng H. X. Qin Y. C. Yan A. L. Xie L. L. (2008). Transfer of iodine from soil to vegetables by applying exogenous iodine. Agron. Sustain. Dev. 28, 575–583. 10.1051/agro:2008033

[B17] Hung C. C. Wong G. T. F. Dunstan W. M. (2005). Iodate reduction activity in nitrate reductase extracts from marine phytoplankton. Bull. Mar. Sci. 76, 61–72.

[B18] Johns T. Eyzaguirre P. B. (2007). Biofortification, biodiversity and diet: a search for complementary applications against poverty and malnutrition. Food Pol. 32, 1–24. 10.1016/j.foodpol.2006.03.014

[B19] Kiferle C. Gonzali S. Holwerda H. T. Ibaceta R. R. Perata P. (2013). Tomato fruits: a good target for iodine biofortification. Front. Plant Sci. 4:205. 10.3389/fpls.2013.00205 23818889 PMC3694224

[B20] Landini M. Gonzali S. Perata P. (2011). Iodine biofortification in tomato. J. Plant Nutr. Soil Sci. 174, 480–486. 10.1002/jpln.201000395

[B21] Mackowiak C. L. Grossl P. R. (1999). Iodate and iodide effects on iodine uptake and partitioning in rice (*Oryza Sativa* L.) grown in solution culture. Plant Soil 212, 135–143. 10.1023/A:1004666607330 11762382

[B22] Mackowiak C. L. Grossl P. R. Cook K. L. (2005). Iodine toxicity in a plant-solution system with and without humic acid. Plant Soil 269, 141–50. 10.1007/s11104-004-0401-6

[B23] Perring L. Basic-Dvorzak M. Andrey D. (2001). Colorimetric determination of inorganic iodine in fortified culinary products. Analyst 126, 985–988. 10.1039/b102423j 11478660

[B24] Piatkowska E. Kopeć A. Biezanowska-Kopeć R. Pysz M. Kapusta-Duch J. Koronowicz A. A.. (2016). The impact of carrot enriched in iodine through soil fertilization on iodine concentration and selected biochemical parameters in wistar rats. PLoS ONE 11:e0152680. 10.1371/journal.pone.0152680 27043135 PMC4820277

[B25] Renna M. Serio F. Signore A. Santamaria P. (2014). The yellow–purple polignano carrot (*Daucus Carota* L.): a multicoloured landrace from the puglia region (Southern Italy) at risk of genetic erosion. Gen. Res. Crop Evol. 61, 1611–1619. 10.1007/s10722-014-0155-9

[B26] Signore A. (2016). Mapping and sharing agro-biodiversity using open data kit and google fusion tables. Comp. Elec. Agric. 127, 87–91. 10.1016/j.compag.2016.06.006

[B27] Signore A. Serio F. Santamaria P. (2016). A targeted management of the nutrient solution in a soilless tomato crop according to plant needs. Front. Plant Sci. 7:391. 10.3389/fpls.2016.00391 27242804 PMC4876364

[B28] Smolen S. Skoczylas Ł. Ledwozyw-Smolen I. Rakoczy R. Kopeć A. Piatkowska E.. (2016). Biofortification of carrot (*Daucus Carota* L.) with iodine and selenium in a field experiment. Front. Plant Sci. 7:730. 10.3389/fpls.2016.00730 27303423 PMC4882318

[B29] Smolen S. Wierzbinska J. Sady W. Kołton A. Wiszniewska A. Liszka-Skoczylas M. (2015). Iodine biofortification with additional application of salicylic acid affects yield and selected parameters of chemical composition of tomato fruits (*Solanum Lycopersicum* L.). Sci. Hortic. 188, 89–96. 10.1016/j.scienta.2015.03.023

[B30] Smolen S. Sady W. Ledwozyw-Smolen I. Strzetelski P. Liszka-Skoczylas M. Rozek S. (2014). Quality of fresh and stored carrots depending on iodine and nitrogen fertilization. Food Chem. 159, 316–322. 10.1016/j.foodchem.2014.03.024 24767061

[B31] Weng H. X. Weng J. K. Yan A. L. Hong C. L. Yong W. B. Qin Y. C. (2008a). Increment of iodine content in vegetable plants by applying iodized fertilizer and the residual characteristics of iodine in soil. Biol. Trace Elem. Res. 123, 218–28. 18265951 10.1007/s12011-008-8094-y

[B32] Weng H. X. Yan A. L. Hong C. L. Xie L. L. Qin Y. C. Cheng C. Q. (2008b). Uptake of different species of iodine by water spinach and its effect to growth. Biol. Trace Elem. Res. 124, 184–194. 10.1007/s12011-008-8137-4 18449478

[B33] White P. J. Broadley M. R. (2009). Biofortification of crops with seven mineral elements often lacking in human diets-iron, zinc, copper, calcium, magnesium, selenium and iodine. New Phytol. 182, 49–84. 10.1111/j.1469-8137.2008.02738.x 19192191

[B34] Winger R. J. König J. House D. A. Konig J. House D. H. (2008). Technological issues associated with iodine fortification of foods. Trends Food Sci. Technol. 19, 94–101. 10.1016/j.tifs.2007.08.002

[B35] Wong G. T. F. Hung C. C. (2001). Speciation of dissolved iodine: integrating nitrate uptake over time in the oceans. Contin. Shelf Res. 21, 113–128. 10.1016/S0278-4343(00)00086-8

[B36] World Health Organization (2005). Preventing Chronic Diseases: A Vital Investment. Available online at: http://www.who.int/entity/chp/chronic_disease_report/contents/part1.pdf?ua=1

[B37] World Health Organization (2007). Assessment of the Iodine Deficiency Disorders and Monitoring Their Elimination. Geneva: WHO.

[B38] Zimmermann M. B. (2007). Interactions of vitamin a and iodine deficiencies: effects on the pituitary-thyroid axis. Int. J. Vit. Nutr. Res. 77, 236–240. 10.1024/0300-9831.77.3.236 18214025

[B39] Zimmermann M. B. (2017). Iodine, in Nutrition and Health in a Developing World, eds de Pee S. Taren D. Bloem M. (Cham: Springer International Publishing), 287–295. 10.1007/978-3-319-43739-2_12

[B40] Zimmermann M. B. Andersson M. (2012). Update on iodine status worldwide. Curr. Opin. Endocrinol. Diab. Obes. 19, 382–387. 10.1097/MED.0b013e328357271a 22892867

